# Dispensing practices issue shorter prescription lengths compared with non-dispensing practices: a quasi-experimental cross-sectional study

**DOI:** 10.3399/BJGPO.2025.0115

**Published:** 2026-02-25

**Authors:** Ian Holdroyd, Liam Loftus, Cameron Appel, Efthalia Massou, John Ford

**Affiliations:** 1 Wolfson Institute of Population Health, Queen Mary University of London, London, UK; 2 Department of Public Health and Primary Care, University of Cambridge, Cambridge, UK

**Keywords:** general practice, primary health care, health sector reform, prescriptions

## Abstract

**Background:**

Dispensing practices, which have in-house dispensaries, are paid to dispense medications directly to patients. Evidence suggests that this financial incentive influences prescribing behaviour, although the underlying mechanisms remain unclear.

**Aim:**

To investigate the impact of dispensing status on prescription length in England.

**Design & setting:**

Quasi-experimental, repeated-measures, cross-sectional study of English general practices.

**Method:**

Business administration data classified practices as dispensing or non-dispensing, determined the percentage of patients eligible for dispensing, and captured prescription lengths for seven drugs with fixed dosing regimens at 3-month intervals from July 2023 to April 2024. Generalised estimating equations analysed the relationship between dispensing status and the proportion of patients eligible for dispensing with average prescription length, controlling for patient and practice characteristics.

**Results:**

Adjusting for patient and practice characteristics, dispensing practices prescribed shorter average lengths for all drugs. Desogestrel showed the largest difference (21.9 days shorter), followed by indapamide standard release (9.85 days), indapamide modified release (9.71 days), ezetimibe (8.41 days), tamsulosin (7.18 days), alendronic acid (6.63 days), and dapagliflozin (5.85 days). An increase in the proportion of patients eligible for dispensing was associated with significantly shorter prescription lengths across all drugs. Dispensing practices more consistently prescribed medications for 28–31 days, whereas non-dispensing practices showed greater variability.

**Conclusion:**

Dispensing practices are associated with shorter prescription lengths, increasing the number of prescriptions issued over time and the associated dispensing fee. The absence of clear guidance on prescription lengths likely contributes to this variability. Central bodies should consider providing explicit recommendations to optimise prescription durations.

## How this fits in

Dispensing practices in the UK have higher prescribing costs, in part owing to a greater number of prescriptions and a higher proportion of expensive medications. International evidence suggests they issue shorter prescriptions to maximise dispensing fees, but no studies have examined the impact of dispensing status on average prescription length in England. Our quasi-experimental analysis shows that dispensing practices consistently prescribe shorter prescriptions across all drug categories. Moreover, the stronger the financial incentive, the greater the difference in prescribing behaviour.

## Introduction

Prescribing costs in NHS primary care totalled £10.1 billion in 2023–2024, accounting for 49.0% of the overall NHS prescribing budget.^1^ Given the significant financial pressures on the NHS, it is crucial to ensure that all spending — particularly a budget as substantial as prescription costs — is managed as efficiently as possible.

Primary care medications are either dispensed by community pharmacies or in-house dispensaries within general practices. Practices with in-house dispensaries are referred to as dispensing practices. As of April 2024, 922 practices in England (14.8%) operated as dispensing practices. Strict criteria govern eligibility of which patients can be dispensed to: primarily patients living more than 1.6 km from a pharmacy in approved, typically rural areas are eligible. As of 2024, for each prescription dispensed, practices receive a tariff fee, reflecting the cost of the drug minus a clawback (ranging from 3.17%–11.18%), plus a fixed dispensing fee of £2.08–£2.35.^2^ Advocates suggest that these arrangements enhance access to medications in isolated areas and provide financial support to practices in remote rural areas, where the cost of delivering health care is higher.

Some, however, suggest that this results in competing interests, given that the choice of prescription will impact the practice’s profitability.^3–5^ Empirical evidence underscores this: prescribing costs in English dispensing practices are higher than in non-dispensing practices.^4,5^ Similar findings are reported internationally.^6–8^ One study estimated that prescribing differences in dispending practices resulted in £53 million extra in prescribing fees to the NHS.^3^ Studies of English practices suggest that this may be owing to higher prescription rates of high-cost drugs^4,5^ and higher quantity of prescription.^4,9^

Another proposed explanation, supported by international evidence, is that dispensing practices may issue shorter prescriptions to maximise the number of times that they receive a fixed dispensing fee when patients collect medications.^6^ Data in the English context is limited. Older, and local data from Lincolnshire in 1990–1991 found that dispensing practices were more likely to prescribe shorter prescriptions.^9^ In English studies, evidence of smaller prescription quantities has been identified;^4^ however, such data does not account for differences in medication types or dosing, limiting its utility in accurately reflecting prescribing behaviour.

A recent study, which analysed data from all English practices between December 2018 and November 2019, found that dispensing practices were more likely to prescribe shorter 28-day prescriptions rather than 2 or 3-month prescriptions, compared with non-dispensing practices.^10^ Prescription length was estimated based on the number of tablets prescribed for five commonly dispensed medications presumed to be taken once daily: ramipril, atorvastatin, simvastatin, levothyroxine, and amlodipine. These drugs were selected on the assumption of once-daily dosing, and the analysis combined data across all five drugs to compare prescribing patterns between dispensing and non-dispensing practices.

This analysis builds on previous work and strengthens the existing evidence on the relationship between dispensing status and prescription length in four key ways. First, the proportion of dispensing patients within a dispensing practice can vary substantially; for example, from less than 1% to over 99%. Earlier studies in England have compared dispensing and non-dispensing practices as distinct groups; this approach may overlook meaningful variation within practices. By leveraging variation in the proportion of dispensing-eligible patients as a continuous variable, this study adopts a quasi-experimental design that offers much stronger conclusions to be drawn. Second, prior research has often relied on assumptions about dosing regimens to infer prescription lengths from tablet counts, which can introduce bias if dosing varies or split dosing is used. To address this, we apply a systematic approach to drug selection to improve the accuracy of prescription length estimation. Third, recognising that prescription lengths can differ markedly between drugs, we calculate and analyse prescription durations separately for each medication examined. Finally, in addition to examining the distribution of prescription durations, we quantify differences by calculating mean prescription lengths.

## Method

### Study design

We conducted a repeated-measures, quasi-experimental, cross-sectional study, examining all general practices in England across four time points: July 2023, October 2023, January 2024, and April 2024.

### Data sources

We obtained a comprehensive range of practice and prescribing data at each time point for all GP practices in England. Data from the NHS Business Services Authority included the number of patients eligible for dispensing at each practice, total practice list size, and details of all dispensed prescription linked to the prescribing GP practice.^11^ For each prescription, the following information was used: GP practice; drug; drug strength; ‘quantity’ (total unit doses issued across all items, for example, number of tablets); and ‘items’ (the number of times that each specific combination of a drug, dose, and quantity was issued at each practice).

Additionally, deprivation scores for each practice were obtained from the Department for Health and Social Care’s Fingertips dataset, using the 2019 Index of Multiple Deprivation (IMD) score linked to the practice’s postcode.^12^ Age profile data for each practice at each time point was sourced from NHS England Digital.^13^

All data were linked by practice code.

### Included drugs and variables

For drugs with variable dosing regimens, it is not possible to determine whether a larger quantity of tablets (for example, 56 tablets) reflects a longer prescription duration (for example, one 10 mg tablet once daily for 56 days), a different dosing schedule (for example, one 10 mg tablet twice daily for 28 days), or a higher dose (for example, two 10 mg tablets once daily for 28 days). Only medications with fixed dosing regimens have a fixed relationship between quantity and prescription length and therefore could be included. To calculate prescription length accurately and consistently, we therefore developed a systematic approach to identify such appropriate drugs. We identified the 100 most prescribed drugs in England during the study period. Each drug was screened using the *British National Formulary (BNF)* — a pharmaceutical reference containing monographs for all drugs licensed in the UK — to exclude those with variable dosing regimens.

Seven drugs were identified as having a single, standard dosing regimen, allowing for a fixed relationship between quantity prescribed and prescription duration. These were as follows: alendronic acid; dapagliflozin; desogestrel; ezetimibe; indapamide modified release; indapamide standard release; and tamsulosin. For each, all formulations were included, encompassing both branded products and different routes of administration.

### Variables

At each time point, practices were categorised as either dispensing or non-dispensing, and the proportion of patients eligible for dispensing within each practice was calculated. The age profile of each practice was used to determine the median age.

For each drug, the expected total prescription length (in days) was calculated using the prescribed drug strength, quantity, and known daily dose. A mean prescription length for each of the seven included drugs was then calculated for each practice at each time point.

Table 1 shows the variables that were obtained for each practice.

**Table 1.** Data used in analysis

Variables	Time points
Dispensing status (yes or no)	July 2023, October 2023, January 2024, April 2024
Proportion of patients who could be dispensed to	July 2023, October 2023, January 2024, April 2024
Total list size	July 2023, October 2023, January 2024, April 2024
Proportion of patients who were male	July 2023, October 2023, January 2024, April 2024
Median age of patients	July 2023, October 2023, January 2024, April 2024
IMD score	2019
Mean length of prescription for the following drugs: alendronic acid, dapagliflozin, desogestrel, ezetimibe, indapamide (modified release), indapamide (standard release), and tamsulosin	July 2023, October 2023, January 2024, April 2024

IMD = Index of Multiple Deprivation

### Statistical analysis

Simple descriptive analysis was conducted, categorised by dispensing and non-dispensing practices.

For each drug, generalised estimating equation (GEE) models examined the association between practice dispensing status and mean prescription length. A quasi-experimental approach was employed to analyse dispensing practices exclusively, assessing the relationship between the proportion of patients eligible for dispensing and prescription lengths. All models were constructed both unadjusted and adjusted for the following four key variables: Index of Multiple Deprivation (IMD); practice list size; proportion of patients who are male; and median age.

All analyses were conducted in R (version 4.3.2).

## Results

### Practice characteristics

Complete data were available for 6317 practices across the four time points. To ensure dataset completeness, the practice numbers at each time point were compared with an independent national dataset (NHS Workforce Dataset).^13^ Complete data were available for 6313 practices (99.8%) in July 2023, 6285 practices (99.9%) in October 2023, 6271 practices (99.9%) in January 2024, and 6232 practices (99.6%) in April 2024.

Table 2 presents the simple descriptive data for dispensing and non-dispensing practices as of April 2024. Of the 922 dispensing practices in April 2024, 256 had fewer than 20% of patients eligible for dispensing, 250 had between 20% and 40%, 144 between 40% and 60%, 85 between 60% and 80%, and 187 between 80% and 100%.

### Dispensing practices issue shorter average prescription lengths

**Table 2.** Practice characteristics and prescription lengths as of April 2024. Prescription lengths represent the mean prescription lengths for each practice

	All practices	Dispensing practices	Non-dispensing practices
Practice characteristics			
Number of practices	6232	922	5310
Practice list size, mean (SD)	10 147.82 (6900.82)	10 302.96 (7304.74)	10 120.88 (6828.62)
Median age, mean (SD)	41.49 (6.42)	48.55 (5)	40.26 (5.81)
Percentage male patients, mean (SD)	50.33 (2.66)	49.35 (1.18)	50.5 (2.81)
IMD			
1st quintile (most deprived)	1207 (19.4%)	6 (0.7%)	1201 (22.6%)
2nd quintile	1871 (30.0%)	115 (12.5%)	1756 (33.1%)
3rd quintile	1526 (24.5%)	322 (34.9%)	1204 (22.7%)
4th quintile	1275 (20.5%)	393 (42.6%)	882 (16.6%)
5th quintile (least deprived)	352 (5.6%)	86 (9.3%)	266 (5%)
Rurality			
Rural	1053 (16.9%)	677 (73.4%)	376 (7.1%)
Urban	5176 (83.1%)	245 (26.6%)	4931 (92.9%)
Prescription length (days)			
Alendronic acid, mean (SD)	34.55 (6.7)	29.27 (2.96)	35.47 (6.74)
Dapagliflozin, mean (SD)	29.52 (7.76)	25.94 (4.39)	30.14 (8.05)
Desogestrel, mean (SD)	119.45 (36.25)	103.67 (31.02)	122.21 (36.4)
Ezetimibe, mean (SD)	33.18 (9.52)	27.87 (4.52)	34.11 (9.86)
Indapamide modified release, mean (SD)	37.42 (11.03)	30.61 (5.11)	38.63 (11.35)
Indapamide standard release, mean (SD)	36.34 (9.88)	29.04 (5)	37.61 (9.97)
Tamsulosin, mean (SD)	34.13 (8.74)	29.25 (4.45)	34.98 (9.02)

IMD = Index of Multiple Deprivation

Table 3 presents the results of GEE models examining the effects of dispensing status on prescription length. Across all seven drugs, dispensing practices issued shorter prescriptions compared with non-dispensing practices. Dispensing practices issued shorter prescriptions across all seven drugs compared with non-dispensing practices. Adjusted for IMD, median age, list size, and patient sex, the greatest difference was observed for desogestrel (21.9 days), followed by indapamide standard release (9.85 days), indapamide modified release (9.71 days), ezetimibe (8.41 days), tamsulosin (7.18 days), alendronic acid (6.63 days), and dapagliflozin (5.85 days). This corresponds to differences of 17.9%, 26.2%, 25.1%, 24.7%, 20.5%, 18.7%, and 19.4%, respectively. Shorter prescription lengths were also associated with higher deprivation scores and a greater proportion of male patients.

**Table 3.** Association of dispensing status and average prescription length

	Alendronic acid	Dapagliflozin	Desogestrel	Ezetimibe	Indapamide modified release	Indapamide standard release	Tamsulosin
Whether practice is a dispensing practice (unadjusted)	-5.75 (-6.10 to -5.41)	-4.01 (-4.40 to -3.63)	-17.36 (-19.49 to -15.22)	-6.04 (-6.49 to -5.6)	-7.96 (-8.40 to -7.51)	-8.03 (-8.54 to -7.53)	-5.09 (-5.58 to -4.60)
Whether practice is a dispensing practice (adjusted)	-6.63 (-7.08 to -6.18)	-5.85 (-6.39 to -5.31)	-21.92 (-24.50 to -19.35)	-8.41 (-9.02 to -7.80)	-9.71 (-10.31 to -9.11)	-9.85 (-10.53 to -9.18)	-7.18 (-7.88 to -6.47)
10-point increase in IMD score	-2.28 (-2.41 to -2.14)	-1.61 (-1.78 to -1.43)	-1.96 (-2.79 to -1.13)	-2.78 (-2.99 to -2.56)	-2.74 (-2.99 to -2.50)	-2.62 (-2.83 to -2.41)	-2.19 (-2.39 to -1.99)
1000 person increase in list size	0.02 (0.00 to 0.05)	0.05 (0.02 to 0.08)	0.73 (0.57 to 0.88)	0.02 (-0.02 to 0.05)	-0.01 (-0.05 to 0.02)	0.00 (-0.03 to 0.03)	0.04 (0.00 to 0.07)
1% increase in percentage of patients who are male	-0.09 (-0.15 to -0.03)	-0.21 (-0.30 to -0.13)	-1.5 (-1.86 to -1.14)	-0.26 (-0.36 to -0.15)	-0.13 (-0.27 to 0.00)	-0.33 (-0.42 to -0.23)	-0.30 (-0.41 to -0.19)
1 year increase in median age	-0.17 (-0.21 to -0.14)	0.02 (-0.02 to 0.06)	0.12 (-0.05 to 0.30)	-0.06 (-0.11 to -0.01)	-0.12 (-0.17 to -0.07)	-0.12 (-0.17 to -0.08)	-0.02 (-0.07 to 0.02)

Results of generalised estimating equation (GEE) analysis investigating the effects of dispensing status. Beta coefficients, and 95% confidence intervals are shown for each variable. Effect sizes are measured in prescription length in days. IMD = Index of Multiple Deprivation

Table 4 summarises the effects of a higher proportion of dispensing-eligible patients within dispensing practices. For all seven drugs, a higher proportion of dispensing-eligible patients was linked to shorter average prescription lengths. The largest difference was observed for desogestrel (1.71 days per 10% increase in eligible patients), followed by indapamide standard release (0.43 days), ezetimibe (0.41 days), indapamide modified release (0.33 days), tamsulosin (0.39 days), dapagliflozin (0.31 days), and alendronic acid (0.27 days).

**Table 4.** Association of the proportion of dispensing-eligible patients and average prescription length

	Alendronic acid	Dapagliflozin	Desogestrel	Ezetimibe	Indapamide modified release	Indapamide standard release	Tamsulosin
10% increase in proportion of patients for dispensing (unadjusted)	-0.31 (-0.38 to -0.25)	-0.38 (-0.49 to -0.28)	-2.48 (-3.00 to -1.96)	-0.46 (-0.56 to -0.36)	-0.37 (-0.47 to -0.26)	-0.51 (-0.62 to -0.39)	-0.44 (-0.56 to -0.31)
10% increase in proportion of patients for dispensing (adjusted)	-0.27 (-0.35 to -0.20)	-0.31 (-0.42 to -0.20)	-1.71 (-2.31 to -1.11)	-0.41 (-0.52 to -0.29)	-0.33 (-0.45 to -0.21)	-0.43 (-0.56 to -0.30)	-0.39 (-0.53 to -0.25)
10 point increase in IMD score	-0.24 (-0.55 to 0.06)	-0.39 (-0.85 to 0.06)	-0.97 (-4.49 to 2.55)	-0.67 (-1.14 to -0.20)	-0.45 (-1.01 to 0.12)	-0.38 (-0.93 to 0.17)	-0.67 (-1.14 to -0.20)
1000 person increase in list size	0.01 (-0.02 to 0.05)	0.04 (0.00 to 0.09)	0.21 (-0.13 to 0.56)	0.02 (-0.04 to 0.07)	0 (-0.05 to 0.05)	0.05 (0.00 to 0.11)	0.04 (-0.01 to 0.10)
1% increase in percentage of patients who are male	-0.01 (-0.13 to 0.10)	-0.12 (-0.32 to 0.08)	0.63 (-0.85 to 2.10)	0.08 (-0.13 to 0.29)	0 (-0.24 to 0.23)	-0.02 (-0.24 to 0.20)	0.05 (-0.13 to 0.24)
1 year increase in median age	-0.04 (-0.08 to 0.01)	-0.04 (-0.11 to 0.02)	-0.91 (-1.31 to -0.51)	-0.06 (-0.14 to 0.02)	-0.06 (-0.14 to 0.01)	-0.03 (-0.11 to 0.05)	-0.01 (-0.09 to 0.06)

Results of generalised estimating equation (GEE) analysis investigating the effects of the proportion of patients in dispensing practices that area eligible to be dispensed to. Beta coefficients, and 95% confidence intervals are shown for each variable. Effect sizes are measured in prescription length in days. IMD = Index of Multiple Deprivation

### Dispensing practices issue more 1-month long prescriptions compared with non-dispensing practices

Table 5a and b display the distribution of prescription lengths for all included medications, stratified by dispensing and non-dispensing practices. Dispensing practices were less likely to issue prescriptions shorter than 28 days. Conversely, non-dispensing practices exhibited greater variability, with a broader distribution across both shorter and longer durations. Notably, desogestrel had longer prescription lengths. Desogestrel is prescribed as a contraceptive medication to younger patients, who are less likely to be on other medications. Therefore, a monthly prescription would present a considerable barrier to such patients, given that they are much less likely to need to collect other medications.

**Table 5.** Lengths of prescriptions in (a) dispensing and (b) non-dispensing practices

a. Days prescribed (dispensing practices)	Alendronic acid	Dapagliflozin	Desogestrel	Ezetimibe	Indapamide modified release	Indapamide standard release	Tamsulosin
0–7	0%	12.6%	1.6%	9.5%	5.5%	5.4%	10.2%
8–27	0%	4.3%	0.3%	0.3%	0.3%	0.4%	0.3%
28–31	96.1%	79.2%	7.9%	84.6%	88.1%	87.7%	84.5%
32–45	0%	0%	0%	0%	0%	0%	0%
46–60	3.7%	3.7%	0.7%	5.3%	5.7%	6.2%	4.8%
≥61	0.2%	0.1%	89.5%	0.3%	0.3%	0.3%	0.3%
Total	100%	100%	100%	100%	100%	100%	100%
**b. Days prescribed (non-dispensing practices)**	**Alendronic acid**	**Dapagliflozin**	**Desogestrel**	**Ezetimibe**	**Indapamide modified release**	**Indapamide standard release**	**Tamsulosin**
0–7	0%	22.5%	1.7%	19.3%	13.4%	12.4%	18.2%
8–27	0%	3.7%	0.4%	0.4%	0.4%	0.5%	0.3%
28–31	77.5%	52.0%	8.9%	52.4%	51.8%	52.6%	53.1%
32–45	0%	0.1%	0%	0%	0%	0%	0%
46–60	20.7%	20.3%	1.3%	25.2%	31.1%	31.5%	26%
≥61	1.7%	1.4%	87.7%	2.7%	3.3%	3%	2.4%
Total	100%	100%	100%	100%	100%	100%	100%

Proportion of all prescriptions in dispensing (5a) and non-dispensing (5b) practices that were prescribed for between 0–7 days, 8–27 days, 28–31 days, 32–45 days, 46–60 days, or for >61 days. Prescription lengths represent every individual prescription issued rather mean values.

## Discussion

### Summary

Dispensing practices were more likely to be found in more affluent areas and in rural areas. They had an older population but no significant difference in their list size. Dispensing practices prescribe medications for shorter durations compared with non-dispensing practices. A quasi-experimental approach demonstrated that a higher proportion of dispensing-eligible patients within a practice correlated with shorter prescription lengths. This offers stronger evidence of a causal relationship as the greater the proportion of dispensing-eligible patients, the greater the financial incentive to issue shorter prescriptions. This difference is attributable to dispensing practices issuing more consistent prescription durations, predominantly in the 28–31 day range. Notably, shorter prescription lengths in dispensing practices were not driven by very brief durations (for example, 1 week or less), as such prescriptions were less common in these practices.

### Strengths and limitations

The primary strength of this study lies in its methodological approach. It employs a quasi-experimental design, conducts separate analyses for each drug, and uses a rigorous screening process to minimise inaccuracy and bias when calculating prescription lengths.

This study analyses seven drugs, which may not fully represent prescribing patterns across all medications. Desogestrel, alendronic acid, and ezetimibe were affected by supply shortages during the study period, which may have influenced prescribing behaviour. Nonetheless, the structured approach to drug selection ensures accuracy in calculating prescription durations and substantially strengthens the study’s design. The analysis draws on a comprehensive national dataset, comprising 11 153 159 prescriptions. This large and representative sample further reinforces the robustness and generalisability of the findings.

While the cross-sectional design increases the risk of confounding and restricts causal inference, this study is bolstered by robust control of multiple confounding factors and a quasi-experimental approach, enhancing the ability to draw stronger causal conclusions.

Our analysis encompassed all GP practices in England across multiple time points, ensuring extensive coverage. Although some data loss occurred owing to incomplete information from specific practices — a common challenge with linked datasets — data completeness remained exceptionally high at each time point, reinforcing the reliability of the results.

### Comparison with existing literature

Consistent with previous findings, this study identified that dispensing practices prescribe shorter durations of medication.^6,9,10^ This prescribing behaviour contributes to higher costs associated with prescribing in dispensing practices, a phenomenon widely reported both in England and internationally.^4–9^ A Swiss study highlighted that increased prescription frequency was the most significant driver of elevated prescription costs.^6^ Other mechanisms contributing to higher costs in dispensing practices include the prescription of more non-generic and higher-cost medications,^4,5^ as well as increased overall prescribing rates.^4,9^

A recent study of English practices found that dispensing practices were more likely to prescribe 28-day prescriptions compared with non-dispensing practices, investigating five commonly prescribed medications.^10^ Our findings build on this work by quantifying differences in average prescription lengths for each drug. We included a larger set of drugs, applied more rigorous inclusion criteria, and calculated prescription lengths separately for each drug to account for variation across medications. By examining the relationship between the proportion of dispensing-eligible patients and average prescription length, our quasi-experimental approach provides a more nuanced and robust contribution to the evidence base. In comparison to this previous study, we also included prescriptions with durations of <1 month. This allows for a more complete characterisation of prescribing behaviours. Notably, shorter prescriptions often reflect specific circumstances — such as the use of monitored dosage systems (for example, Dosette boxes) — that may fall outside the prescriber’s direct control. Dispensing practices were less likely to issue very short prescriptions (Table 5). Had we excluded these prescriptions as in previous studies, the estimated difference in prescription lengths between dispensing and non-dispensing practices would have been greater.

### Implications for research and practice

This study considerably strengthens the evidence available regarding the impact of dispensing status on prescription length. The stronger quasi-experimental approach may suggest the influence of economic incentives on GP prescribing behaviours, whereby dispensing practices are more likely to adopt consistent prescribing behaviours when incentivised to do so. This analysis alone does not clarify the extent of financial benefit for dispensing practices nor the impact on patient care. Additional work would be helpful to further develop understanding of the impact of dispensing practices on prescription lengths, costs, and patient outcomes. This could include qualitative interviews, more holistic cost and cost-effectiveness analysis, and data on patient outcomes.

Local policies often recommend repeat medications being 28-days long; however, this widespread adoption of 28-day prescriptions is based on outdated, non-systematic research.^14–16^ A recent systematic review has challenged this practice, citing insufficient evidence to justify it and calling for its reconsideration.^14^ The review highlighted moderate-strength evidence that longer prescription durations improve medication adherence, while evidence suggesting increased medication waste was inconsistent and of low quality. Furthermore, longer prescriptions are associated with lower overall costs when dispensing fees and prescriber time are taken into account.^17^ This finding is particularly pertinent for medications prescribed for long-term, stable conditions, such as those examined in this study. Reflecting this, some integrated care boards (ICBs) have begun recommending 56-day prescriptions for patients on stable, non-complex regimens.^18^

Currently, there is a lack of specific guidance on appropriate prescription lengths. The Department of Health and Social Care’s 2007 guidance advises that prescription lengths should *‘balance patient convenience with clinical appropriateness, cost-effectiveness, and patient safety*’.^19^ Given the potential cost savings and absence of clear direction,the Department of Health and Social Care should consider issuing explicit recommendations on prescription lengths, particularly for chronic and stable conditions.

## Data

Data available upon reasonable request.
